# Ovarian cyst fluid is a rich proteome resource for detection of new tumor biomarkers

**DOI:** 10.1186/1559-0275-9-14

**Published:** 2012-12-27

**Authors:** Björg Kristjansdottir, Karolina Partheen, Eric T Fung, Janusz Marcickiewicz, Christine Yip, Mats Brännström, Karin Sundfeldt

**Affiliations:** 1 Department of Obstetrics and Gynecology, Institute of Clinical Sciences, Sahlgrenska Cancer Center, University of Gothenburg, S-413 45, Gothenburg, Sweden; 2 Vermillion, Inc., Fremont, CA, USA

**Keywords:** Biomarkers, Cyst fluid, Epithelial ovarian cancer, Ovarian cyst, Proteomic

## Abstract

**Background:**

We aimed to investigate the use of ovarian cyst fluid as a source for biomarker discovery and to find novel biomarkers for use in the diagnosis of epithelial ovarian tumors.

**Results:**

Ovarian cyst fluids from 218 women were collected and 192 (benign *n* = 129, malignant *n* = 63) were analyzed using mass spectrometry. 1180 peaks were detected, 221 of which were differently expressed between benign and malignant ovarian tumors. Seventeen peaks had receiver operating curve and area under the curve values >0.70; the majority of these represented peaks for apolipoproteins C-III and C-I (ApoC-I), transthyretin (TTR), serum amyloid A4 (SAA4), and protein C inhibitor (PCI). ApoC-III, PCI, and serum CA125, with an ROC AUC 0.94 was the best combination for diagnosing epithelial ovarian cancer. ApoC-III and PCI was analyzed with ELISA in the original cohort (*n* = 40) and in 40 new cyst fluid samples for confirmation with an independent method and validation. Results from MS and ELISA for ApoC-III correlated well (*p* = 0.04). In the validation set, ApoC-III was significantly (*p* = 0.001) increased in the malignant epithelial ovarian cancers.

**Conclusions:**

Fluid from ovarian cysts connected directly to the primary tumor harbor many possible new tumor-specific biomarkers. Biomarkers found in ovarian cyst fluid may be used as molecular imaging targets for early diagnostics and prediction of therapy. Plasma abundant proteins are also influencing the cystic fluid proteome. Methods for isolating less frequent cyst fluid proteins are needed.

## Background

The incidence of ovarian cancer in Europe and the United States is 11/100 000, and about 200 000 women worldwide are diagnosed with the disease each year [1]. More than 70% of all ovarian cancers are detected in the late stages (FIGO III-IV), and the five-year survival rate is only 25–30% for these patients. If, however, ovarian cancer is detected in stage I, when the disease is still confined to the ovary, the five-year survival rate is over 85% [2]. Neither reliable diagnostic tests nor accurate imaging techniques are yet available to distinguish between benign and malignant cysts. Improvement in the diagnosis of this lethal disease is very desirable. Serum biomarkers for the early detection of EOC such as CA125 and HE4 have shown promising results in women with a pelvic mass [3,4], but CA125 shows false negative results in approximately 40% of stage I tumors [5]. HE4 complements CA125 especially in younger women, were CA125 can be false positive in a variety of physiological conditions and benign diseases, including endometriosis [6]. These markers are not sensitive enough for the reliable detection of small volume disease, and complementation with other markers is required.

Over the past twenty years, different strategies have been applied to search for new biomarkers as diagnostic tools for the early detection of EOC. One approach is genomics with RNA expression array-based research; others focus on proteins using MS and ELISA [7]. Serum is the most common source in the search for new biomarkers, but proteomic profiles of urine and ascites have also been evaluated [8-11]. Because the blood proteome contains contributions from all organ systems in the body, an alternative approach is the proteomic analysis of a proximal fluid, such as ductal or cystic fluids in breast- or pancreatic cancer, and in our case cyst fluids from the ovary [12]. Ovarian tumors commonly grow in cystic formations, regardless of histology. A cyst can become large enough to reach the diaphragm and still be benign, while a stage III cancer with ascites and peritoneal carcinomatosis may present with relatively small changes in the ovaries. The ovarian cyst fluid probably represents the local microenvironment, containing proteins secreted directly by ovarian tumor cells, surrounding stroma and other cells involved in the tumorigenesis. Early pathological changes within this organ may therefore be reflected in proteomic patterns found in ovarian cyst fluid before secretion into the blood stream. If so, biomarkers in the cyst fluid may be used as direct targets for new diagnostic imaging techniques in early stage ovarian cancer. Few discoveries of markers for diagnostics and prognosis have been reported in studies of ovarian cyst fluid [13-16], but ovarian cysts, especially those that are malignant, have higher concentrations of proteins than diluted serum and urine [15].

In this study, we collected 218 cyst fluid samples, 192 were diagnosed with benign, borderline type, and malignant ovarian tumors of different histology and stages and analyzed with SELDI-TOF MS. Five most significant peaks were identified and a peak combination with highest ROC AUC was evaluated. ELISA was used to confirm and validate protein expression found by SELDI-TOF MS in the original sample set and a new set of cyst fluids. CA125 was analyzed in corresponding serum samples from our original cohort and used in the ROC AUC calculations. Our aim was to analyze the prospect of cyst fluid as a potential source of biomarkers for ovarian tumors.

## Results

### Proteomic profiling

#### Seventeen significant peaks had ROC AUC values >0.70

**Figure 1:**
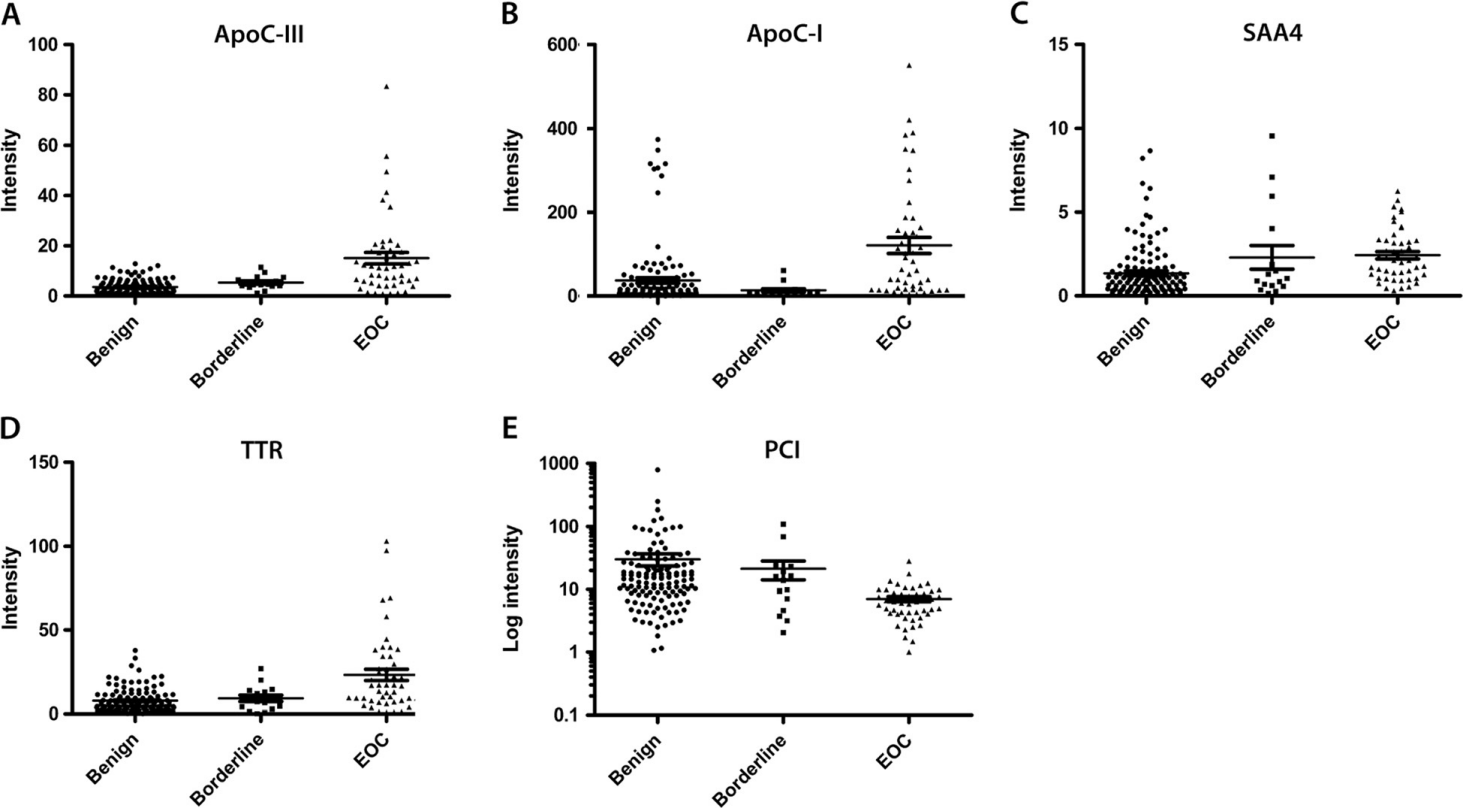
**Peak intensity in cyst fluids from benign, borderline type, and malignant ovarian tumors (n = 192).** (**A**) ApoC-III; (**B**) ApoC-I; (**C**) SAA4; (**D**) TTR; (**E**) PCI.

A total of 1180 peaks were resolved by SELDI-TOF MS, and of these, 221 peaks were differently expressed (*p* <0.0001) in benign and malignant samples. Several peaks in the cyst fluid were identified as albumin and other proteins that also are abundant in blood [17]. Seventeen peaks had ROC AUC values of >0.70 when comparing benign and malignant cases (Table 1). Of these 17 peaks, 5 peaks were identified as apoliprotein C-III (ApoC-III), 3 peaks as apolipoprotein C-I (ApoC-I), 2 peaks as transthyretin (TTR), 1 peak as serum amyloid A4 (SAA4), and 1 peak as protein C inhibitor (PCI; SerpinA5) (see methods section). A comparison of peak levels in fluid from benign and borderline cysts showed no significant differences for any of the five peaks. Due to the small group size borderline tumors (*n* = 16), were not further evaluated, but their peak levels are shown in the scatter plots for each protein (Figure 1A-E). The study population was between 16–86 years old, median age in the benign cohort was relatively high, almost the same as for the malignant cohort (Table 2). Analysis of peak levels correlation with age showed that protein levels were not influenced by patients’ age (data not shown). There was high percentage of stage I + II (50%) in the original population of women with ovarian cysts (Table 2).

**Table 1:** Significantly (p<0.0001) different mass peaks between benign and malignant cyst fluid samples, with ROC AUC >0.70 are listed with m/z value and identified peaks

**Protein**	**Fraction**	**Peak m/z value**	**Mean intensity benign/malignant**	**ROC AUC**	**Specificity % (CI)**
**PCI**	EB-E3	3902	30.23 / 7.00	0.79	67.7 (58.9-75.6)
**ApoC-III**	EB-E3	9743	3.60 / 15.09	0.82	68.5 (59.7-76.3)
ApoC-III	EB-E3	9448	5.29 / 22.00	0.80	60.8 (51.8-69.2)
ApoC-III	EB-E2	9751	4.65 / 24.2	0.80	60.8 (51.8-69.2)
ApoC-III	EB-E2	9777.5	3.20 / 14.7	0.79	63.1 (54.2-71.4)
ApoC-III	EB-E3	9453	7.30 / 33.20	0.78	50 (41.1-58.9)
**ApoC-I**	MEP-M1	6647	37.53 / 121.14	0.78	57.7 (48.7-66.3)
ApoC-I - truncated	MEP-M1	6448	68.91 / 184.53	0.76	53.1 (44.1-61.9)
ApoC-I - truncated	MEP-M1	6489	24.49 / 52.75	0.76	53.1 (44.1-61.9)
**SAA4**	MEP-M1	12886	1.35 / 2.42	0.76	58.5 (49.5-67.0)
SAA4	MEP-M1	12863	1.53 / 2.72	0.74	59.2 (50.3-67.8)
**TTR**	EB-E3	13900	7.80 / 23.16	0.77	59.2 (50.3-67.8)
TTR	EB-E3	13925	5.36 / 14.27	0.75	57.7 (48.7-66.3)
Hb beta	FT	8037	2.61 / 4.64	0.75	65.4 (56.5-73.5)
Albumin	FT	54622	0.73 / 0.30	0.76	61.5 (52.6-69.9)
Albumin	FT	54426	1.72 / 0.25	0.75	60.8 (51.8-69.2)
Albumin ion	MEP-M1	44754	1.06 / 0.50	0.76	57.7 (48.7-66.3)

PCI = protein C inhibitor, ApoC-III = apolipoprotein C-III, ApoC-I = apolipoprotein C-I, SAA4 = serum amyloid A4, and TTR = transthyretin, EB = Equalizer Beads, differentially eluted yield fractions E2, and E3, MEP = Mercapto ethyl pyridine beads, differentially eluted yield fractions M1.

**Table 2:** Sample characteristics of the original cohort including age, histology, stage (FIGO), and grade (without excluded samples, n = 26)

**Total**	**Benign**	**Borderline**	**Malignant**	**Stage**				**Grade**		
**n = 192**	**n = 129**	**n = 16**	**n = 47**	**I n = 21**	**II n = 3**	**III n = 20**	**IV n = 3**	**High n = 17**	**Moderate n = 14**	**Poor n = 16**
**Mean Age** (range)	**60** (16-86)	**56** (40-85)	**61** (40-85)							
	*n* (%)	*n* (%)	*n* (%)							
**Simple**	33 (26)									
**Stromal**	9 (7)									
**Endometrioma**	9 (7)									
**Hemorrhagic**	3 (2)									
**Dermoid**	5 (4)									
**Serous**	45 (35)	9 (56)	30 (64)	11	2	15	2	8	11	11
**Mucinous**	25 (19)	7 (44)	6 (13)	4		1	1	5	1	
**Endometrioid**			5 (11)	4	1			2	1	2
**Clear cell**			3 (6)	1		2		2	1	
**Undifferentiated**			3 (6)	1		2				3

#### Peaks for Apo C-III and PCI in combination - independent predictor of malignancy

**Figure 2:**
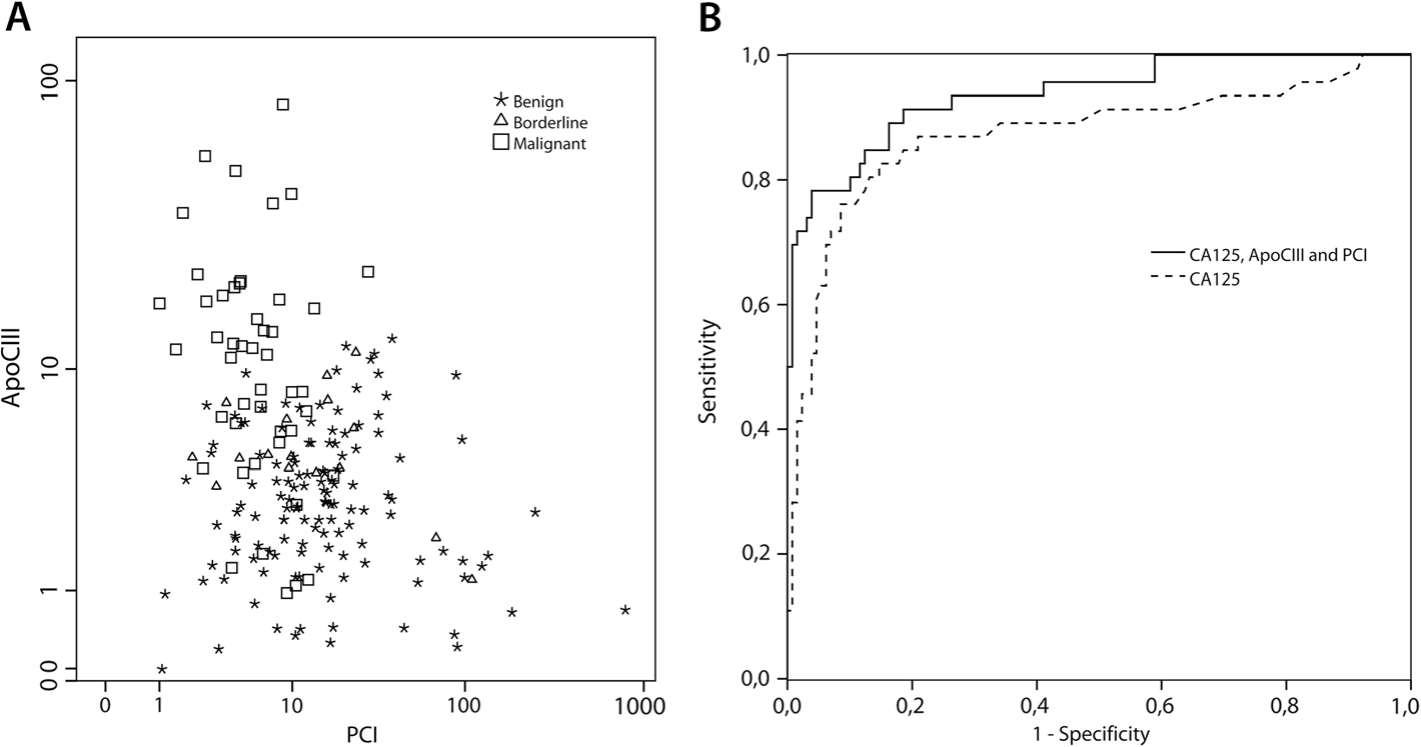
(A) Scatter plot of logarithmic values of the two independent markers, ApoC-III and PCI; (B) ROC AUC of CA125 only and of CA125 combined with ApoC-III and PCI.

The peaks with highest ROC AUC for each protein in cyst fluid (PCI, ApoC-III, ApoC-I, SAA4 and TTR) were selected for further statistical analysis together with corresponding serum CA125. ROC AUC for serum CA125 was 0.87 (CI 0.80-0.94). Combined ROC AUC for the five cyst fluid markers was 0.91 (CI 0.86-0.96). Logistic regression analyze showed that peaks for ApoC-III (*p* <0.0001) and PCI (*p* = 0.001) were independent factors in predicting malignancy (Figure 2A). These two markers together achieved ROC AUC of 0.91 (CI 0.85-0.96), the same as for the 5 markers ROC AUC. Marker panel of the peaks for ApoC-III, PCI, and serum CA125 generated the highest ROC AUC of 0.94 (CI 0.89-0.98) (Figure 2B). The specificity of this three-marker panel was 88.4% compared to 68.2% for CA125 alone. CA125 showed a sensitivity of 81.8% using cut-off 35 U/ml, accordingly specificity was calculated for the peaks with highest ROC AUC at fixed sensitivity of 81.8%; 68.5% and 67.7% for Apo-CIII and PCI respectively (Table 1). None of the peaks showed superior specificity to that of CA125 when analyzed alone.

### Confirmation and validation - ELISA

This study was primarily conducted to explore ovarian cyst fluid as a possible source of new biomarkers for detection and diagnosis of ovarian cancer. With SELDI-TOF MS we found 221 peaks to be differently expressed between benign and malignant ovarian cyst fluid. However the suggested proteins for the peaks with best marker combination was chosen for confirmation of method and validation in an external sample set.

**Figure 3:**
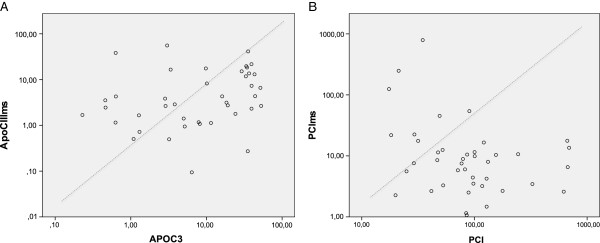
Scatter plot of correlations between SELDI-TOF MS (peaks) and ELISA (proteins) for the identified (A) ApoC-III (Spearman’s rho 0,338; p = 0.04) but not (B) PCI (Spearmans’s rho -0.255; p = 0.11).

Peaks for ApoC-III and PCI, which had the highest ROC AUC in the MS analysis, were analyzed with ELISA in 40 ovarian cyst fluid samples (Table 3) from the original cohort to evaluate their existence with an independent method. Results from the two different methods, SELDI-TOF MS and ELISA, correlated for ApoC-III, Spearman’s rho = 0.328; *p* = 0.04 (Figure 3A). Significant (*p* < 0.05) increase of ApoC-III (ELISA) in the malignant compared to the benign ovarian cysts was also found (Table 4). PCI, however, was increased (ELISA) in the malignant ovarian cysts and decreased in the MS analyze, which did obviously not correlate, (Spearman’s rho -0.255; *p* = 0.11) (Figure 3B). The increase of PCI in malignant compared to benign ovarian cyst fluids was not significant (Table 4). Next, we analyzed a new set of 40 consecutively collected ovarian cyst fluid samples (Table 3) for ApoC-III and PCI protein levels with ELISA (Table 4). Median ApoC-III was 3.3 in benign compared to 22.7 in malignant ovarian cyst fluids, which was found to be significant (*p* = 0.001). Median PCI (active antigen) was 4.7 in benign and 14.9 in malignant ovarian cyst fluids, which was not significant (*p* = 0.26).

**Table 3:** Characteristics of the samples from the original cohort and new samples including histology and stage (FIGO), used in the ELISA confirmation and validation of SELDI-TOF MS results

	**Benign**	**Stage I**	**Stage II**	**Stage III**	**Malignant**
Original cohort:	n = 20:	n = 12:	n = 0:	n = 8:	n = 20:
New samples	n = 20	n = 7	n = 1	n = 12	n = 20
**Simple**	4:6				
**Stromal**	1:1				
**Endometrioma**	2:1				
**Serous**	8:8	6:2	0:1	6:11	12:14
**Mucinous**	5:4	3:1		1:0	4:1
**Endometrioid**		2:2			2:2
**Clear cell**		1:2		1:0	2:2
**Undifferentiated**				0:1	0:1

**Table 4:** Confirmation and validation with an independent method (ELISA) of MS peaks with the highest ROC AUC combination (0.91)

**Protein**	**Confirmation set**			**Validation set**		
	**Benign**	**Malignant**	**Sign.**	**Benign**	**Malignant**	**Sign.**
	Median	Median		Median	Median	
	(range)	(range)		(range)	(range)	
**APOC3**	**5.16**	**13.1**	**p < 0.05**	**3.3**	**22.7**	**p = 0.001**
	(0.2-52.8)	(0.6-51.7)		(0.04-35.2)	(1.8-377.0)	
**PCI**	**52.8**	**100.4**	**NS**	**4.7**	**14.9**	**NS**
	(17.5-177.7)	(20.0-691.8)		(0.0-332.9)	(0.0-196.2)	

## Discussion

The aim of the present study was to analyze the prospect of cyst fluid as a source for biomarker discovery. We detected numerous differently expressed protein peaks in our samples, showing that proteomic analysis of ovarian cyst fluids may serve as a valuable tool in the ongoing quest for new ovarian cancer biomarkers. Biomarkers produced by the tumor are most likely to be found close to the tumor in the initial phase, before excretion into the blood [12]. The existence of a protein in cyst fluid can, however, also depend on diffusion from the blood stream. The peaks with highest ROC AUC separating benign from malignant ovarian cyst corresponded to proteins that are also present in blood [17]. Even if some of these proteins are not directly expressed by the tumors, they may reflect reactions that are closely related to carcinogenesis, such as increased thrombotic activity and inflammation, and may thus still be candidates as markers for tumor detection.

MS techniques allow rapid and high throughput analysis of the entire proteome in a small biological sample. One disadvantage with large scale screening methods using serum samples is the dominance of highly abundant proteins. Ninety-five percent of the serum consists of only 20 abundant proteins i.e. albumin, immunoglobulins, transferrin and macroglobulins [18]. By using ovarian cystic fluid we made the assumption that we would increase the chance for detection of rare, more tumor-specific proteins. However, there seem to be a high resemblance between ovarian cyst fluid and the serum proteome since the abundant large proteins were the most prominent ones also in the present study. Similar resemblances between serum and fluids from other compartments such as pancreatic cysts, ascites and breast duct fluids have also been described [8,11,12]. Use of careful and selective depletion of high abundance proteins from the ovarian cyst fluid might increase access to other specific biomarkers. One major limitation with the method used in the present study is the lack of exact protein identification of the mass-peaks. Fortunately the abundant peaks observed as differentially expressed between benign and malignant ovarian cystic fluid, have been purified and confidently identified in several earlier studies [19-27]. Especially the peaks for ApoC-I and TTR have been correctly identified after several different preanalytic conditions [27]. To further validate ovarian cyst fluids as a source for biomarker discovery of rare but specific proteins, depletion must be performed and a MS with accurate protein identification i.e isobaric tags for relative and absolute quantification (iTRAQ) could be used.

We compared the protein peak profiles in cysts from benign and malignant ovarian tumors and detected peaks corresponding to five potential biomarkers. In combination with serum CA125, two mass peaks were found to be predictors of malignancy (ROC AUC 0.94). The first peak with the suggested protein, ApoC-III, is a very low-density lipoprotein, a carrier protein for diverse proteins, mainly synthesized in the liver and small intestines. ApoC-III inhibits lipoprotein lipase and hepatic lipase and plays an important role in regulating the triglyceride rich lipoproteins and thereof regulates many cellular functions. Our study is the first to describe increased ApoC-III in ovarian cyst fluids. In the analysis of an eleven protein panel in serum from patients with ovarian cancer, increased Apo-CIII was one of the markers that could distinguish women with cancer from those with benign conditions [28]. The second peak with the suggested protein, PCI (SerpinA5), is a component of the anticoagulant protein C pathway, originally identified in human plasma as an inhibitor of activated protein C. PCI also inhibits urinary plasminogen activator (uPA), which is a mediatior of tumor cell invasion [29]. PCI is expressed in various human fluids and tissues but has not previously been described in ovarian cyst fluids. Gene and protein expression analysis revealed SERPINA5, the gene encoding PCI, as down-regulated in serous ovarian carcinomas compared with serous borderline tumors, which is consistent with our MS results [30,31]. SERPINA5 expression is decreased in renal and prostate cancer and elevated levels have been related to better survival in breast cancer [29,32,33]. However, although we found a significant decrease of PCI levels in the malignant compared to the benign cohort using SELDI-TOF MS we could not confirm our MS results with an independent method, ELISA. This suggests that the peak we identified as PCI were incorrect identified. However, due to the various functions and versatility of PCI it is challenging to investigate PCI protein expression in body fluids [34]. In blood PCI is found in various forms including inactive, cleaved, and complex-bound. PCI is also modified through post-translational events and has been reported as microheterogeneous as a result of differences in N-glycan structures [35].

The other three peaks with ROC AUC > 70 identified with mass matching from the literature in this study was TTR, SAA4 and ApoC-I. TTR formerly called prealbumin, has previously been identified as a potential early diagnostic marker for ovarian cancer and known as a marker for acute-phase inflammatory response and nutritional status [22,24]. A study of ovarian tumors showed a direct correlation between serum levels, intracellular expression of SAA4 and malignant state of the epithelial cells [36]. In colon cancer SAA4 levels increases gradually from epithelial dysplasia to cancer in tissue samples and are highest in metastatic cancer, which supports the possibility of its role in carcinogenesis [37]. In our study, SAA4 was up-regulated in ovarian cyst fluid from patients with malignant tumors, which is consistent with validated SELDI-TOF MS in serum from ovarian cancer patients [26]. The peak for ApoC-I was also increased in the malignant ovarian cysts in our study. Takano et al. [38] have shown that ApoC-I is abundantly expressed in pancreatic neoplastic epithelium. It was also detected in the culture medium of a pancreatic cancer cell line, which suggests that cancer cells secrete ApoC-I. The elevated levels of ApoC-I in our analysis may also depend on secretion of the protein from tumor cells.

The ultimate goal is to find superior biomarkers to those already known to detect early EOC. Our group and others evaluated CA125 and HE4 in blood from patients with pelvic tumors. In the study by Moore et al. the ROC AUC for CA125 was 0.83 compared to our 0.87 and in combination CA125 with HE4 0.91 and 0.85 respectively [3,4]. Bearing in mind that the selected peaks in this study has not been analyzed for exact peptide sequence we still choose to perform a comparative estimation of ROC AUC between the benign and malignant samples. None of the protein peaks detected in our study showed a better specificity than CA125 when considered in isolation. However, a combination of the peaks for ApoC-III, PCI, and CA125 improved the ROC AUC significantly. The currently presented marker combination of serum CA125 and two cystic fluid mass-peaks (ROC AUC 0.94) was comparable to the above-mentioned algorithms [3,4]. Despite the drawbacks of the method used in our present study, our results strengthen our hypothesis that it is possible to find new biomarkers in the ovarian cystic fluid that can diagnose early ovarian cancer.

## Conclusion

We demonstrate for the first time that ovarian tumor cyst fluid is a promising source for the detection of novel diagnostic biomarkers with proteomic profiling. Fluid from ovarian cysts connected directly to the primary tumor harbor many possible new tumor-specific biomarkers. Biomarkers found in ovarian cyst fluid may be used as molecular imaging targets for early diagnostics and prediction of therapy.

## Methods

### Clinical samples

Cyst fluids were collected prospectively and consecutively from 218 women presenting with a suspected ovarian tumor cyst during the five years from March 2001 to September 2006 (Table 2). Patients were diagnosed by transvaginal sonography or computed tomography and admitted for surgical removal of the cyst by skilled gynecologic oncology surgeons at Sahlgrenska University Hospital, Gothenburg, Sweden. According to our protocol, blood samples were taken after anesthesia but before surgery, and ovarian cyst fluids were collected after removal of the cyst from the abdomen. All samples were immediately put in 4°C for 15-30 minutes, centrifuged, and aliquoted into Eppendorf tubes. The fluids were transferred to −80°C, within 30–60 minutes after collection. Samples used in this study had one freeze-thaw cycle.

### Sample characteristics

Of the 218 women originally included in the study, we were unable to analyze nine samples due to their high viscosity and high blood content. These nine samples consisted of four benign lesions, one borderline type tumor; FIGO stage IA, two serous EOC; FIGO stage IA and IIIB and two endometrioid EOC; FIGO stage IIA and IIIC. Three non-epithelial germ cell ovarian cancers and fourteen samples that were found to be metastases from cancers other than EOC were also excluded. CA125 was measured in blood samples from all patients with ovarian disease using ELSA-CA125 (Cisbio Bioassays, France) according to the manufacturer’s instructions. All tumors were examined by an experienced pathologist for diagnosis, histology, and grade (Table 2). The tumors were staged (I-IV) according to FIGO standards (Table 2). The study was approved by the local ethics committee at the University of Gothenburg, and each patient provided their informed written consent.

### Protein expression profiling of ovarian cyst fluid – SELDI-TOF MS

High throughput sample fractionation by tandem Equalizer beads (EB) and Mercapto ethyl pyridine (MEP) beads was used. We deposited 200 μl cyst fluid into each well of a 96-well deep-well plates, and added 50 μl 2 M guanidine thiocyanate, 9 M urea 2% CHAPS, and 50 mM Tris-HCl pH 9. After shaking for 20 minutes, each sample was diluted with 500 μl 50 mM Tris-HCl–containing protease inhibitor cocktail (Roche, USA), and added to 70 μl 50% v/v Equalizer resin bead (custom synthesized hexapeptide libraries, American Peptide, USA) in an FDTS-modified fritted 96-well deep-well filter plate (Nunc, USA). The plate was shaken for 60 minutes and centrifuged to collect the unbound fraction. The whole unbound fraction was transferred to 200 μl 50% v/v MEP Hypercel bead (Pall Life Sciences, USA) in an FDTS-modified fritted 96-well deep-well filter plate (Nunc). The plate was shaken for 20 minutes and centrifuged to collect the flow-through fraction. Each well of the 96-well filter plate was washed with 500 μl 50 mM Tris-HCl 0.04% Triton X100 pH 7.5 four times. Proteins bound to the Equalizer beads were differentially eluted by (1) 100 μl 1 M NaCl, 50 mM Tris-HCl, 0.04% Triton X100 pH 7.5 to yield fraction E1, (2) 100 μl 50% isopropanol/acetonitrile (2:1), 1% formic acid, 2% trifluoroacetic acid to yield fraction E2, (3) 100 μl 8 M guanidine-HCl, 1% Triton X100 (90°C) to yield fraction E3a, and (4) 100 μl 6 M urea, 30% ethylene glycol, 0.1 M sodium carbonate to yield fraction E3b. Proteins bound to the MEP beads were differentially eluted by (1) 150 μl 50% isopropanol/acetonitrile (2:1), 0.5% formic acid, 1% trifluoroacetic acid to yield fraction M1, (2) 150 μl 8 M guanidine-HCl, 1% Triton X100 (90°C) to yield fraction M2a, and (3) 150 μl 6 M urea, 30% ethylene glycol, 0.1 M sodium carbonate to yield fraction M2b. Finally, 20-40 μl aliquot of each eluted fraction was added to 200 μl of buffer in a bioprocessor containing either cation exchange ProteinChip array CM10 (Ciphergen, USA) or immobilized Cu ProteinChip array IMAC30 (Ciphergen). After 45 minutes incubation the arrays were washed with 200 μl buffer, then rinsed with water. Sinapinic acid was added and the chip-bound proteins were profiled with a PCS4000 mass spectrometer (Ciphergen) with data acquisition up to 250 kDa m/z and a matrix cutoff of 1 kDa. Each array was read twice and mass focus was set at 4 kDa for first reading and 15 kDa for second reading, sample rate was set at 400 and spectra were collected by the accumulation of 540 shots in the positive mode. Mass accuracy was calibrated externally using All-in-One Peptide and All-in-One Protein Standards (Ciphergen). Spectral patterns were then compared across samples to find discriminating masses or changes in peak intensities. The obtained spectra were analyzed by Ciphergen Express data manager software with Biomarker Wizard and Biomarker Pattern software. Mass spectra were baseline subtracted and normalized to total ion current intensity. Peaks were detected using a signal-to noise ratio of 5, valley depth of 5, 20% minimum peak threshold of all spectra, and a mass range of 2-200 kDa. The peak clusters were completed by a second mass peak detection using a signal-to-noise ratio of 2, valley depth of 2 and cluster mass window of 1 peak width. All analyses were blinded to the operator.

### Determination of marker identity

A couple of very prominent mass peaks characterize SELDI/MALDI profiles of serum. These peaks primarily represent highly abundant proteins like apolipoprotein and fragments hereof. These abundant proteins can be confidently identified based on mass matching with literature [20,27,39]. Each of the five peaks with ROC AUC values >0.70 in our present study have been purified and identified in separate studies. The two members of the apoplipoprotein family, ApoC-I and ApoC-III, produce significant peaks in SELDI protein profiling. The mass peak with *m/z* 6647 detected with MEP in fraction M1 has been confidently identified as ApoC-I [19-21,25,27] and the mass peak with *m/z* 9448 detected with EB in fraction E3 has been confidently identified as ApoC-III [19,20,25]. The mass peak with *m/z* 13900 detected with EB in fraction E3 has been confidently identified as TTR [22,24,26], the mass peak with *m/z* 12886 detected with MEP in fraction M1 was identified as SAA4 [21,26], and the mass peak with m/z 3902 detected with EB in fraction E3 has been identified as PCI, also known as SerpinA5 [23].

### ELISA

Confirmation and validation samples were analyzed with ApoC-III (Human) ELISA kit (KA0465, Abnova, Taiwan), diluted 1:400 before analysis, and levels were presented as μg/mL. PCI actibind ELISA kit (TC16100 Technoclone, Austria), samples were diluted 1:2 before analysis, and levels were presented as percent of normal pooled plasma. Both assays were run according to manufacturer’s instructions. For confirmation ovarian cystic fluid samples from the original cohort were chosen based on measured peak levels from the whole spectra of low to high levels in the SELDI-TOF MS. For validation a new set of 40 patient samples, consecutively collected to our ovarian cyst fluid biobank (2008–2009), were included (Table 3).

### Statistical analysis

Statistical analyses were performed in SPSS for Windows (version 17), CiphergenExpress, and Prism 5.0 (GraphPad). The Mann-Whitney U-test was used to identify peaks with *p*-values <0.0001 for differences between benign and epithelial ovarian tumors. The peak levels were further subjected to logistic regression analysis to determine whether the cyst fluids could be classified. For each model, ROC curves were constructed and values were calculated for the AUCs. The relation between peak levels and age was evaluated using bivariate Pearson correlation coefficient and the relation between protein levels measured with ELISA and SELDI-TOF MS was evaluated with using bivariate Spearman correlation coefficient. Statistical differences in ELISA protein levels between benign and malignant samples were evaluated using the Mann-Whitney U test and a value of *p* <0.05 was considered to be significant.

## Abbreviations

ApoC-I: Apolipoprotein C-I; ApoC-III: Apolipoprotein C-III; CA125: Cancer antigen 125; EB: Equalizer Beads; EOC: Epithelial ovarian cancer; FIGO: International Federation of Gynecology and Obstetrics; HE4: Human epididymis protein 4; MEP: Mercapto-ethyl-pyridine; PCI: Protein C inhibitor; ROC: Receiver operating curve; SAA4: Serum amyloid A4; TTR: Transthyretin.

## Competing interests

The authors declare the following conflict of interest: E.T. Fung and C. Yip were employed by Vermillion Inc.

## Authors’ contributions

BK - Planning of study, collection of material, ELISA, evaluating data, manuscript writing. KP - Statistical evaluation of data, ELISA, manuscript writing. ETF - SELDI-TOF MS performance, data evaluation. JM - Collection of material, manuscript writing. CY - SELDI-TOF MS performance, data evaluation. MB - Planning of study, collection of material, manuscript writing. KS - Planning of study, collection of material, evaluation and analyses of data, manuscript writing.
